# Pharmacological Inhibition of STING/TBK1 Signaling Attenuates Myeloid Fibroblast Activation and Macrophage to Myofibroblast Transition in Renal Fibrosis

**DOI:** 10.3389/fphar.2022.940716

**Published:** 2022-07-18

**Authors:** Haimei Zeng, Ying Gao, Wenqiang Yu, Jiping Liu, Chaoqun Zhong, Xi Su, Shihong Wen, Hua Liang

**Affiliations:** ^1^ Department of Anesthesiology, Foshan Women and Children Hospital, Foshan, China; ^2^ The First Clinical Medical College, Guangdong Medical University, Zhanjiang, China; ^3^ Department of Anesthesiology, Huidong People’s Hospital, Huizhou, China; ^4^ Department of Anesthesiology, The First People’s Hospital of Foshan, Foshan, China; ^5^ Department of Paediatrics, Foshan Women and Children Hospital, Foshan, China; ^6^ Department of Anesthesiology, The First Affiliated Hospital of SUN YAT-SEN University, Guangzhou, China

**Keywords:** STING, TBK1, renal fibrosis, myeloid fibroblast, macrophage

## Abstract

Renal fibrosis is an important pathological biomarker of chronic kidney disease (CKD). Stimulator of interferon genes/TANK binding kinase 1 (STING/TBK1) axis has been identified as the main regulator of innate immune response and closely related to fibrotic disorder. However, the role of STING/TBK1 signaling pathway in kidney fibrosis is still unknown. In this study, we investigated the effect of pharmacological inhibition of STING/TBK1 signaling on renal fibrosis induced by folic acid (FA). In mice, TBK1 was significantly activated in interstitial cells of FA-injured kidneys, which was markedly inhibited by H-151 (a STING inhibitor) treatment. Specifically, pharmacological inhibition of STING impaired bone marrow-derived fibroblasts activation and macrophage to myofibroblast transition in folic acid nephropathy, leading to reduction of extracellular matrix proteins expression, myofibroblasts formation and development of renal fibrosis. Furthermore, pharmacological inhibition of TBK1 by GSK8612 reduced myeloid myofibroblasts accumulation and impeded macrophage to myofibroblast differentiation, resulting in less deposition of extracellular matrix protein and less severe fibrotic lesion in FA-injured kidneys. In cultured mouse bone marrow-derived monocytes, TGF-β1 activated STING/TBK1 signaling. This was abolished by STING or TBK1 inhibitor administration. In addition, GSK8612 treatment decreased levels of α-smooth muscle actin and extracellular matrix proteins and prevents bone marrow-derived macrophages to myofibroblasts transition *in vitro*. Collectively, our results revealed that STING/TBK1 signaling has a critical role in bone marrow-derived fibroblast activation, macrophages to myofibroblasts transition, and kidney fibrosis progression.

## Introduction

The threat of chronic kidney disease (CKD) on public health has become a great global concern, which leads to severe personal morbidity and mortality and economic cost to the society (Liu et al., 2021a). Hypotension, hypovolemia, cardiac pulmonary bypass, and toxin-drugs administration during intraoperative period are the high-risk factors of acute kidney injury (AKI) (Zarbock et al., 2018; Walker and Bell, 2019). It is widely acceptable that incomplete recovery from AKI can cause long-term renal dysfunction and has been identified as a major contributor to CKD (Zhao et al., 2019). Renal fibrosis is an important pathological biomarker of CKD, which is characterized by accumulation of a large amount of extracellular matrix (ECM) that replaces normal parenchyma, eventually results in end stage renal disease. Currently, therapeutic strategy targeting renal fibrosis remains unsatisfactory. Therefore, significant exploration for molecular mechanism should be given to this devastating disorder in order to developing efficient approaches to interrupt renal fibrosis progression.

Stimulator of interferon genes (STING) is an endoplasmic reticulum dimeric adaptor protein. STING is expressed in various haematopoietic cells, including T lymphocytes, dendritic cells and macrophages, and acts as a major regulator of type I interferon release and innate immune response (Carroll et al., 2016; Srikanth et al., 2019). TANK binding kinase 1 (TBK1) is a cytosolic kinase that is essential for the activation of STING-dependent down-stream signaling. In response to external stimuli, STING translocates from the endoplasmic reticulum to the Golgi, and activates TBK1 (Taguchi et al., 2021). Accumulating data show that STING/TBK1 signaling has been deeply linked with multiple diseases (Lam et al., 2014; Ogawa et al., 2018; Occhigrossi et al., 2021).

Recently, numerous studies have documented that STING or TBK1 plays a pivotal role in fibrotic disorder. H-151, a selective inhibitor of STING, can attenuate cardiac fibrosis following myocardial infarction (Hu et al., 2022). STING activation triggers pro-inflammatory cytokines production and promotes liver fibrosis, while inhibition of STING alleviates liver fibrosis (Shen et al., 2022). Moreover, it is reported that STING deficiency or C-176 (an inhibitor of STING) treatment reduces kidney fibrosis in a mouse model of CKD (Chung et al., 2019). In addition, TBK1 inhibition has a significant anti-fibrotic role in pulmonary and liver fibrosis (Qu et al., 2019; Zhou et al., 2020). Nevertheless, the role of STING/TBK1 pathway and its molecular mechanism in renal fibrosis remains to be further elucidated.

The accumulation of bone marrow-derived myofibroblasts and transition of macrophages to myofibroblasts (MMT) are considered as critical factors that contribute to the pathogenesis of renal fibrosis (Meng et al., 2016; Liang et al., 2017a; Chen et al., 2021a; Liu et al., 2021b; Liu et al., 2021c). Evidences reveal that STING and TBK1 expression in monocyte-derived macrophages is closed related to the development of liver fibrosis in patients with nonalcoholic fatty liver disease (Wang et al., 2020). H-151 inhibits the type I interferon response in bone marrow-derived macrophages (Hu et al., 2022). Based on the above-mentioned findings, we hypothesize that STING/TBK1 signaling plays a role in kidney fibrosis progression through the modulation of bone marrow-derived fibroblasts activation and macrophages to myofibroblasts transition.

In this work, we demonstrated that pharmacological inhibition of STING/TBK1 axis impaired bone marrow-derived fibroblasts activation, impeded macrophages to myofibroblasts transition, and protected against renal fibrosis development in folic acid (FA) nephropathy. Our findings show a critical role of STING/TBK1 signaling in myeloid fibroblasts activation and macrophages to myofibroblasts transition in kidney fibrosis.

## Materials and Methods

### Animals

**FIGURE 1:**
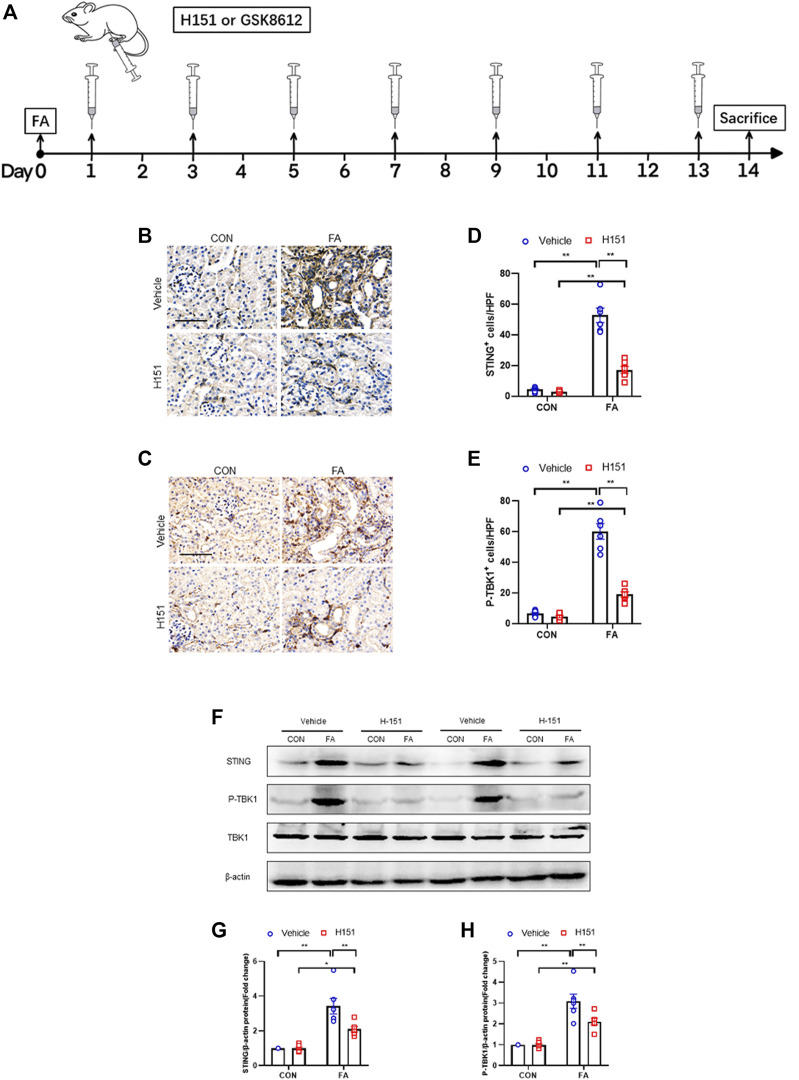
STING/TBK1 signaling pathway is significantly activated in FA nephropathy. **(A)** Timeline diagram of experimental model. **(B,C)** Representative photomicrographs of kidney sections in each group stained for STING (brown) or p-TBK1 (brown). **(D,E)** Quantitative analysis of STING or p-TBK1 positive cells in the kidneys. **(F)** Representative western blots show STING and TBK1 protein levels in the kidneys in each group. **(G,H)** Quantitative analysis of STING and TBK1 protein expression in the kidneys. **p < 0.01, *p < 0.05; *n* = 6 in each group; Scale bar: 50 μm.

Male wild-type (C57BL/6) mice weighing 20–25 g, 8–10 weeks old, were purchased from Guangdong Experimental Animal Center (Guangzhou, Guangdong Province) for experiments. Mice were fed in the laboratory animal house, maintained comfortable temperature, humidity and 12/12-h light/dark cycle, replaced cages and padding daily. FA–induced renal fibrosis was induced by single intraperitoneal injection of 250 mg/kg FA (Sigma). After FA-induced acute kidney injury in mice, 1.5 mg/kg of TBK1 inhibitor (GSK8612) (Jiang et al., 2021) or 7 mg/kg of STING inhibitor (H151) (Gong et al., 2021) was intraperitoneally injected on 1, 3, 5, 7, 9, 11, 13 days after FA treatment. The same volume of DMSO was injected served as control. Kidneys were harvested at 14 days after FA injection (Figure 1A). All animal handling were in accordance with the Animal Ethics Committee of Sun Yat-sen University (No. 2017-692).

### Histology

Kidneys were fixed with 10% buffer formalin, embedded in paraffin and made into paraffin sections (4 μm). Paraffin sections were stained with Hematoxylin-eosin (H&E), Picrosirius red and Masson’s trichrome reagents. The stained sections were observed and imaged by microscope equipped with digital camera (Olympus, Japan) to detect renal injury and fibrosis. Measurement of the fibrotic area was quantified with ImageJ software.

### Immunofluorescence

Frozen sections (5 μm) were fixed with acetone for 10 min, penetrated by 10% Triton for 10 min, and then blocked with 5% goat serum for 1 h. The tissues were incubated overnight with primary antibodies. Rabbit anti-fibronectin (1:200, Abcam), rabbit anti-collagen I (1:200, Abcam), mouse anti-α smooth muscle actin (1:200, Sigma), mouseanti-CD45 (1:100, BD), rabbit anti-α-SMA (1:100, Abcam), rat anti-F4/80 (1:100, Bio-Rad), rabbit anti-α-SMA (1:100, Abcam), rat anti-CD206 (1:100, Bio-Rad), and rabbit anti-α-SMA (1:100, Abcam) at 4 °C. The secondary antibodies of Alexa Fluor 488 goat anti-rabbit (1:400, Abcam), Alexa Fluor 647 goat anti-mouse (1:400, Abcam) and Alexa Fluor 647 goat anti-rat (1:400, Abcam) we reselected to incubate at room temperature for 1 h. Dye the nucleus with DAPI. The images were taken by fluorescence microscope equipped with digital camera (Olympus, Japan) orconfocal laser scanning microscope (Zwiss, Germany). Measurement of the fluorescence staining area was performed using ImageJ software.

### Immunohistochemistry

Paraffin sections were sliced in 4 μm thickness, dehydrated with xylene, rehydrated with gradient alcohol. De-endogenous peroxidase was used to reduce non-specific staining. Antigen retrieval performed by microwave heating in citric acid antigen repair solution (pH = 6.0). All sections were incubated rabbit anti-p-TBK1 (1:100, CST) or rabbit anti-STING (1:100, CST) overnight at 4°C. Detection of bound antibody was carried out using biotinylated goat anti-rabbit secondary antibody, incubated at room temperature for 1 h. The sections were then developed with ABC solution, DAB solution, and counterstained with hematoxylin. The reaction was monitored under a microscope, and terminated in time. The images were taken by microscope image system (Olympus, Japan).Western Blot

RIPA Buffer, protease inhibitor, phosphatase inhibitor, and PMSF according to the ratio were used to configure the lysate and extracted cells and mouse kidney tissue sprotein. The protein concentration was measured by BCA method, added the loading buffer, and subsequent boiled for 10 min at 100°C. Sodium dodecyl sulfate polyacrylamide gel (10 holes) was prepared, and the same amount of protein samples were added to the holes for electrophoresis (60–120 V, 120 min). Rapid membrane transfer solution (SORFA) prepared with ethanol, transferred to PVDF membranes, and closed in blocking solution. Membranes were incubated with STING (1:1000, CST), TBK1 (1:1000, CST), p-TBK1 (1:1000, CST), Fibronectin (1:1000), collagen I (1:1000, MILLIPORE), Periostin (1:1000, GeneTex), α-SMA (1:1000, sigma), β-actin (1:5000, Abclone), overnight at 4°C. Blots were developed using HRP-conjugated appropriate secondary antibodies. Protein bands were analyzed using ImageJ software after the membrane was scanned by automatic Western blotting system (Protein Simple, United States).

### Bone Marrow Monocytes Culture

Bone marrow mononuclear cells were isolated from the femur and tibia of wild-type mice. Cell culture medium was prepared with advanced DMEM, 10% fetal bovine serum, 1% penicillin/streptomycin solution and M-CSF. The cells were cultured in a humidified incubator supplemented with 95% air/5% CO_2_ at 37°C. Cells were stimulated by transformation growth factor-β1 (TGF-β1) (10 ng/ml, Novoprotein) or IL-4 (20 ng/ml, PeproTech) (Chen et al., 2021b) or/and TBK1 inhibitor (GSK8612, 5 μM) (Liu et al., 2021d) for 24 h.

### Statistical Analysis

All data were presented as mean ± SEM, and differences between multiple groups were analyzed by analyzing variance. The Bonferroni procedure in the one-way ANOVA test was used to compare the mean. Differences were considered statistically significant when *p* < 0.05.

## Results

### STING/TBK1 Signaling Pathway Is Significantly Activated in FA Nephropathy

Our findings show that STING-positive cells were profoundly increased in the kidneys of mice following FA treatment, while administration of H-151 markedly decreased these cells in injured kidneys. The STING-positive cells were mainly detected in interstitial cells in the kidneys (Figures 1B,D). In addition, TBK1 was significantly activated in interstitial cells of the kidneys in response to FA stress, which was attenuated by H-151 treatment (Figures 1C,E). In line with the results of immunohistochemical staining, data of western blot show that STING protein levels were increased in FA-injured kidneys. Conversely, H-151 treatment notably down-regulated the expressions of STING protein in injured kidneys. We next discerned whether pharmacological inhibition of STING has an impact on TBK1 activation in protein level. Our data showed that the phospho-TBK1 protein levels were significantly un-regulated in injured kidneys, whereas administration of H-151 led to a considerable reduction of phospho-TBK1 protein levels (Figures 1F–H). These data indicate that STING/TBK1 signaling is significantly activated in FA nephropathy. STING inhibitor is able to potently suppress the activation of STING/TBK1 signaling in fibrotic kidneys with FA injury.

### Pharmacological Inhibition of STING Impaired Bone Marrow-Derived Fibroblast Activation and MMT in FA Nephropathy

**FIGURE 2:**
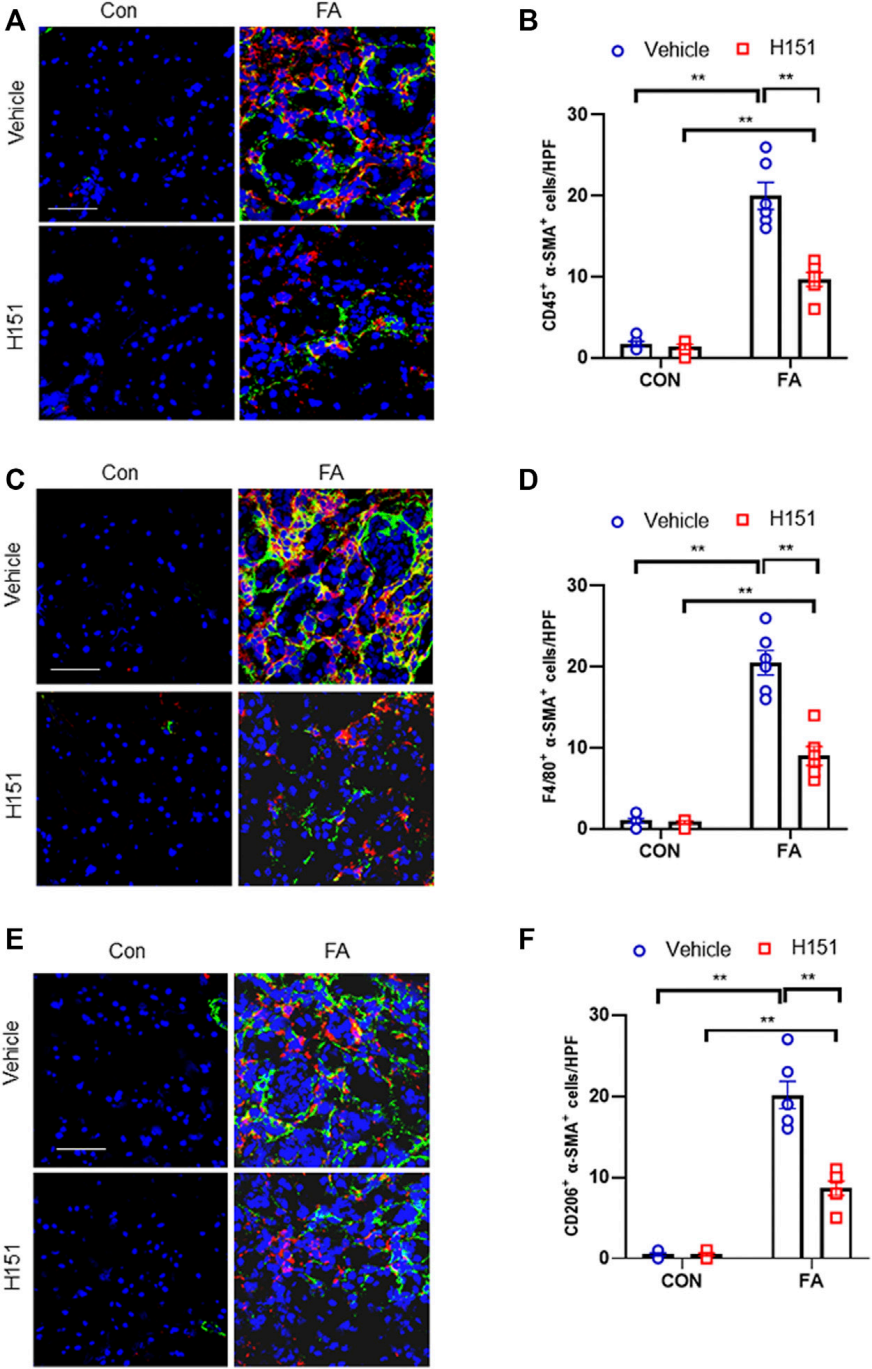
Pharmacological inhibition of STING impaires myeloid fibroblast activation and MMT in FA nephropathy. **(A)** Representative immunofluorescence photomicrographs of kidney sections in each group stained for CD45 (red), α-SMA (green), and DAPI (blue). **(B)** Quantitative analysis of CD45 and α-SMA dual positive cells in the kidneys. **(C)** Representative immunofluorescence photomicrographs of kidney sections in each group stained for F4/80 (red), α-SMA (green), and DAPI (blue). **(D)** Quantitative analysis of F4/80 and α-SMA dual positive cells in the kidneys. **(E)** Representative immunofluorescence photomicrographs of kidney sections in each group stained for CD206 (red), α-SMA (green), and DAPI (blue). **(F)** Quantitative analysis of CD206 and α-SMA dual positive cells in the kidneys. **p < 0.01; n = 6 in each group; Scale bar: 50 μm.

The accumulation of myeloid myofibroblasts and transitions of macrophages to myofibroblasts have an important role in renal fibrosis (Yan et al., 2016; Wang et al., 2017). Data show that STING regulates the activation of bone marrow-derived macrophages *in vitro* (Li et al., 2019). Therefore, we next examined whether STING has a role in myeloid fibroblasts activation in the kidneys with FA injury. Confocal scanning revealed that the number of CD45^+^-α-SMA^+^ dual-positive cells in injured kidneys was significantly increased, whereas pharmacological inhibition of STING by H-151 led to a markedly reduction of these cells in injured kidneys (Figures 2A,B). Furthermore, we showed that FA stress resulted in a sharp elevation of F4/80^+^-α-SMA + cells. By contrast, the number of F4/80^+^-α-SMA + cells in injured kidneys were notable decreased after H-151 treatment (Figures 2C,D). These data suggest that STING has a critical role in myeloid myofibroblasts activation and MMT in FA nephropathy. Evidences show that M2 phenotype macrophages account for the majority of MMT cells (Gong et al., 2021). Thus, we next discerned the transition of M2 macrophages to myofibroblasts in FA-injured kidneys. Our findings reveal that the number of CD206^+^-α-SMA^+^ cells was profoundly increased in kidneys following FA insult. On the contrary, administration of H-151 significantly reduced CD206^+^-α-SMA^+^ cells in kidneys with FA stress (Figures 2E,F). These results indicate that STING plays an important role in differentiation of M2 macrophages into myofibroblasts in FA nephropathy.

### Pharmacological Inhibition of STING Attenuates Progressive Renal Fibrosis in FA Nephropathy

**FIGURE 3:**
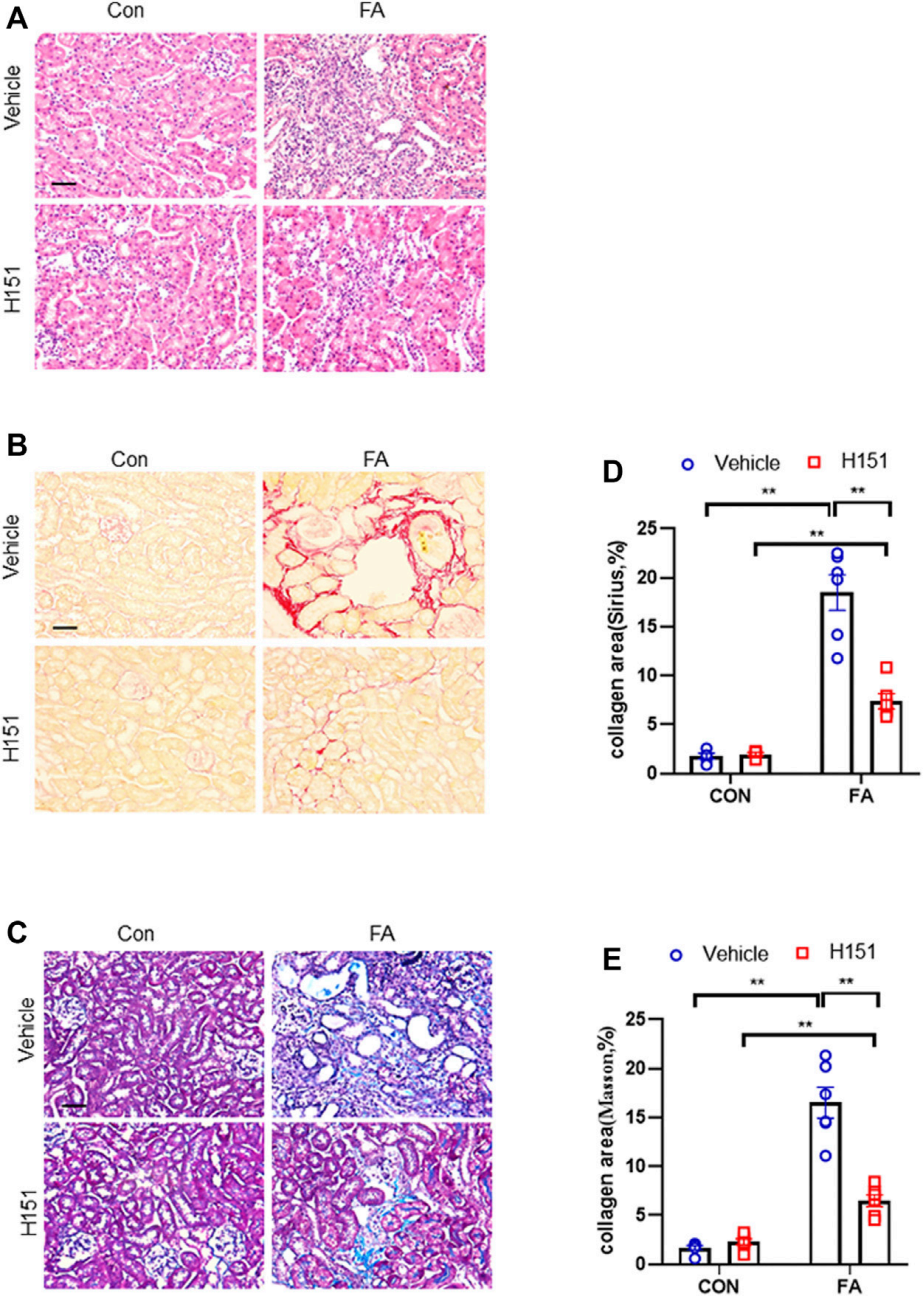
Pharmacological inhibition of STING attenuates progressive renal fibrosis in FA nephropathy. **(A)** Representative photomicrographs of kidneys sections in each group stained with H&E. **(B)** Representative photomicrographs of kidney sections stained with Sirius red. **(C)** Representative photomicrographs of kidney sections stained with Masson’s trichrome. **(D,E)** Quantitative analysis of interstitial collagen area stained with Sirius red or Masson’s trichrome in the kidneys. **p < 0.01; *n* = 6 in each group; Scale bar: 50 μm.

We next examined whether STING inhibitor has an impact on the progression of kidney fibrosis in FA nephropathy. Sirius red, Masson’s trichrome, and H&E staining were used to evaluate fibrotic area or tissue lesion in kidneys of mice subjected to FA treatment. Our findings revealed that FA treatment resulted in a notable increment of the collagen deposition area in injured kidneys of mice. Of note, pharmacological inhibition of STING markedly reduced the collagen area in injured kidneys of mice (Figures 3A–E). These data suggest that pharmacological inhibition of STING suppresses progressive renal fibrosis in FA nephropathy.

### Pharmacological Inhibition of STING Reduces ECM Proteins Expression and Fibroblasts Activation in FA Nephropathy

**FIGURE 4:**
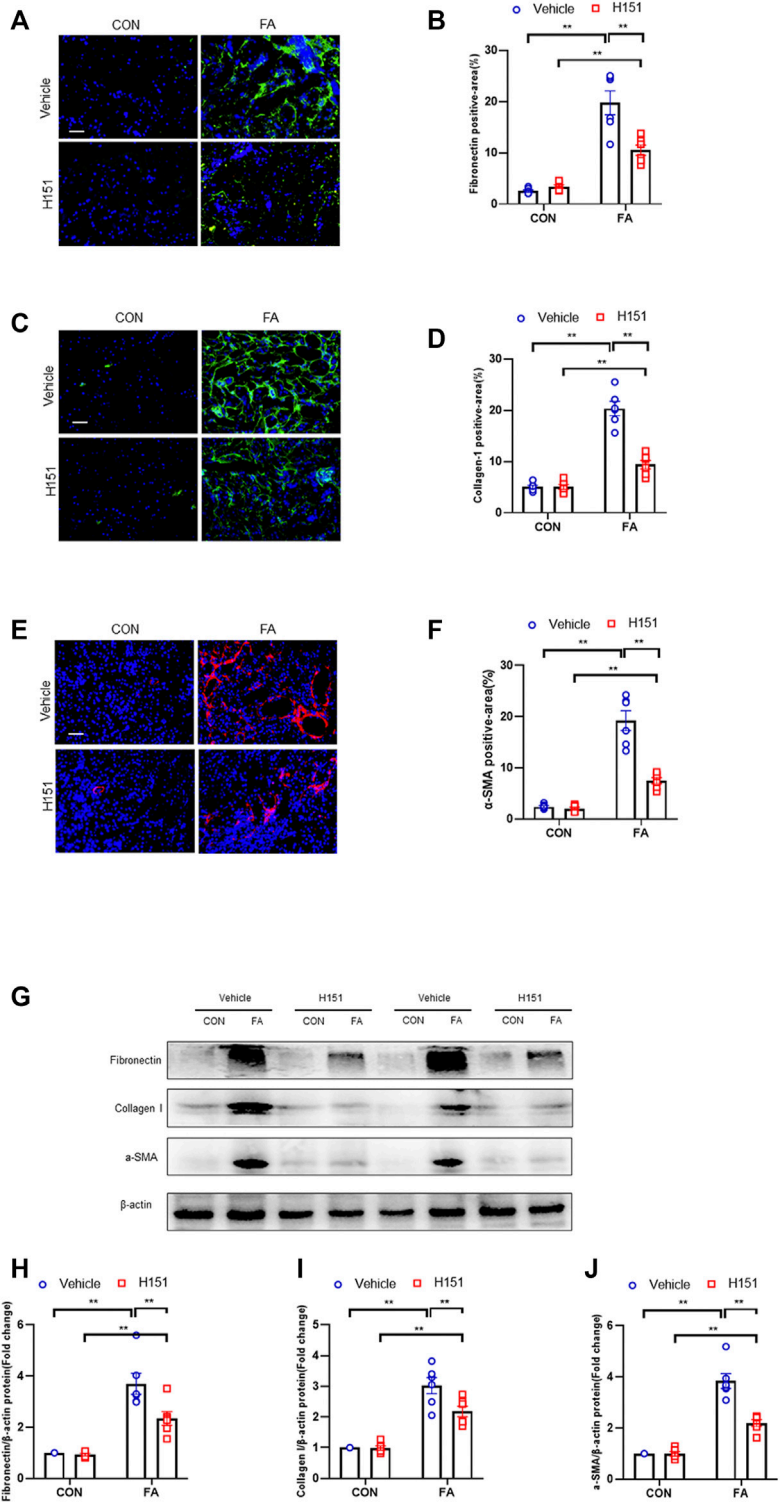
Pharmacological inhibition of STING reduces ECM proteins expression and fibroblasts activation in FA nephropathy. **(A)** Representative immunofluorescence photomicrographs of kidney sections in each group stained for fibronectin (green) and DAPI (blue). **(B)** Quantitative analysis of fibronectin-positive area in the kidneys. **(C)** Representative photomicrographs of kidney sections in each group stained for collagen I (green) and DAPI (blue). **(D)** Quantitative analysis of collagen I-positive area in the kidneys. **(E)** Representative immunofluorescence photomicrographs of kidney sections in each group stained for α-SMA (red) and DAPI (blue). **(F)** Quantitative analysis of α-SMA-positive area in the kidneys. **(G)** Representative western blots show the levels of fibronectin, collagen I, and α -SMA protein expression in each group. **(H–J)** Quantitative analysis of fibronectin, collagen I, and α-SMA protein expression levels in the kidneys. **p < 0.01, *p < 0.05; *n* = 6 in each group; Scale bar: 50 μm.

Kidney fibrosis is characterized by abundant ECM proteins deposition and fibroblasts activation (Liang et al., 2017b). We next assessed the protein levels of fibronectin, collagen I, periostin, and α-smooth muscle actin (α-SMA) in FA nephropathy. Immunofluorescence staining showed that ECM proteins and α-SMA positive area in FA-treated kidneys of mice were profoundly increased. Conversely, administration of H-151 significantly decreased the positive area of these proteins in the kidneys with FA injury (Figures 4A–F). Consistent with the results of immunofluorescence staining, data of western blot showed that pharmacological inhibition of STING significantly down-regulated the protein levels of fibronectin, collagen I, periostin, and α-SMA in injured kidneys of mice (Figures 4G–J). These data suggest that STING inhibition prevents the deposition of ECM proteins and fibroblasts activation in FA nephropathy.

### GSK8612 Treatment Inhibits TBK1 Activation in FA Nephropathy

**FIGURE 5:**
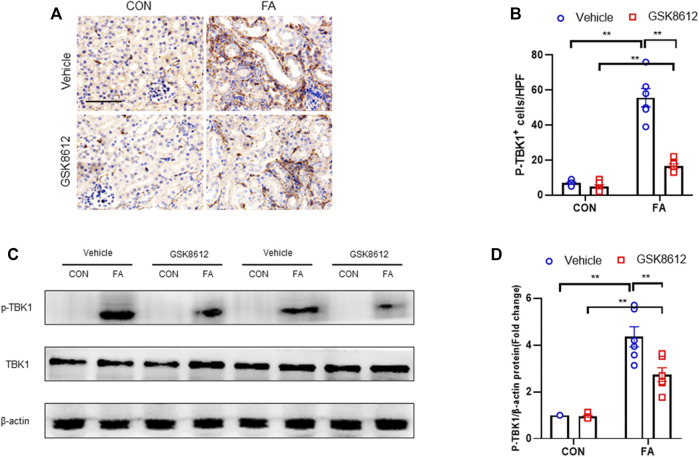
GSK8612 treatment inhibits TBK1 activation in FA nephropathy. **(A)** Representative photomicrographs of kidney sections in each group stained for p-TBK1 (brown). **(B)** Quantitative analysis of p-TBK1 positive cells in the kidneys. **(C)** Representative western blots show TBK1 protein levels in the kidneys in each group. **(D)** Quantitative analysis of TBK1 protein expression in the kidneys. **p < 0.01; *n* = 6 in each group; Scale bar: 50 μm.

We next examined the effect of GSK8612 on the activation of TBK1 in FA nephropathy of mice. We show that FA insult led to a sharp elevation of phospho-TBK1 positive cells number. Of note, pharmacological inhibition of TBK1 by GSK8612 profoundly decreased the number of phospho-TBK1 positive cells in kidneys of mice in response to FA stress (Figures 5A,B). Consistent with afore-mentioned results, administration of GSK8612 notably reduced the protein levels of phoph-TBK1 in injured kidneys (Figures 5C,D). These data indicate that GSK8612 treatment significantly inhibits TBK1 activation in FA nephropathy.

### Pharmacological Inhibition of TBK1 Impeded Myeloid Fibroblast Activation and MMT in FA Nephropathy

**FIGURE 6:**
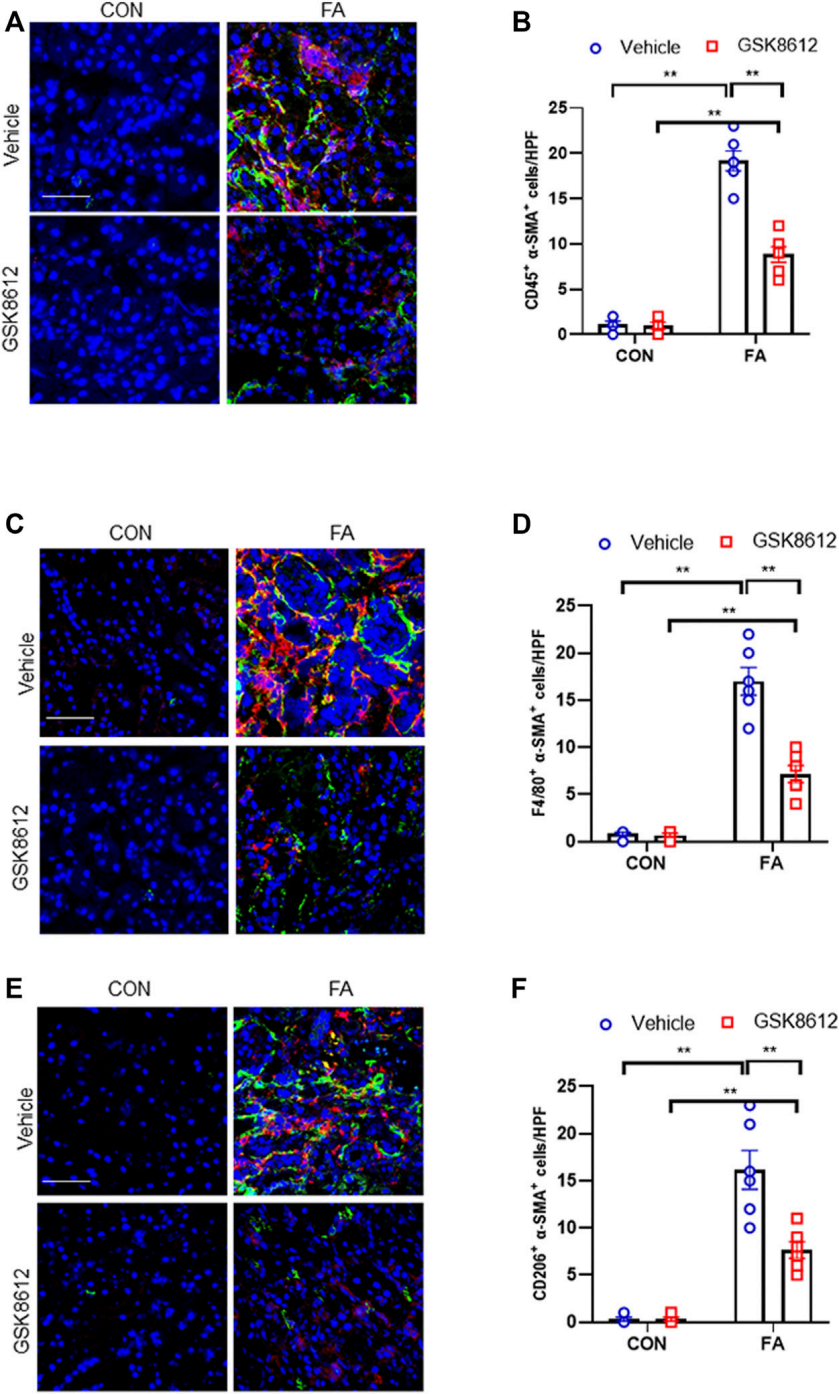
Pharmacological inhibition of TBK1 impedes myeloid fibroblast activation and MMT in FA nephropathy. **(A)** Representative immunofluorescence photomicrographs of kidney sections in each group stained for CD45 (red), α-SMA (green), and DAPI (blue). **(B)** Quantitative analysis of CD45 and α-SMA dual positive cells in the kidneys. **(C)** Representative immunofluorescence photomicrographs of kidney sections in each group stained for F4/80 (red), α-SMA (green), and DAPI (blue). **(D)** Quantitative analysis of F4/80 and α-SMA dual positive cells in the kidneys. **(E)** Representative immunofluorescence photomicrographs of kidney sections in each group stained for CD206 (red), α-SMA (green), and DAPI (blue). **(F)** Quantitative analysis of CD206 and α-SMA dual positive cells in the kidneys. **p < 0.01; *n* = 6 in each group; Scale bar: 50 μm.

To dissect the role of TBK1 in the pathogenesis of kidney fibrosis, we next examined whether pharmacological inhibition of TBK1 prevents myeloid fibroblasts activation and macrophages to myofibroblasts transitions in the kidneys with FA injury. Our findings showed that pharmacological inhibition of TBK1 by GSK8612 caused a profoundly reduction of CD45^+^-α-SMA^+^ cells in injured kidneys as compared with controls (Figures 6A,B). In addition, administration of GSK8612 significantly lowered the number of F4/80^+^-α-SMA^+^ cells in kidneys of mice following FA challenge. Moreover, we revealed that GSK8612 treatment resulted in a considerable reduction of the number of CD206^+^-α-SMA^+^ cells in kidneys with FA injury (Figures 6C–F). These data indicate that TBK1 has a pivotal role in bone marrow-derived fibroblasts activation and macrophages to myofibroblasts transition in FA nephropathy.

### Pharmacological Inhibition of TBK1 Protects Against the Development of Kidney Fibrosis in FA Nephropathy

**FIGURE 7:**
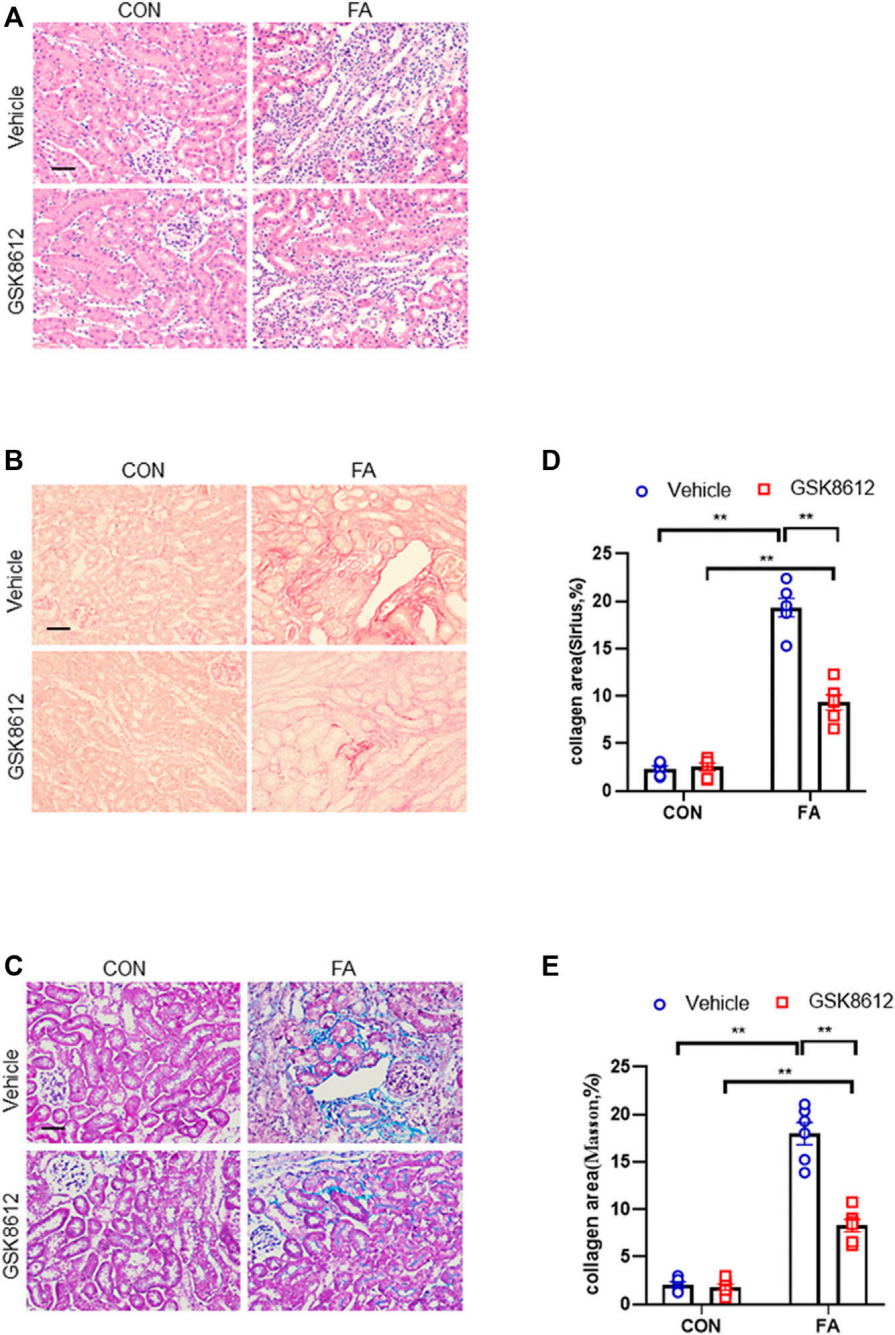
Pharmacological inhibition of TBK1 protects against progressive renal fibrosis in FA nephropathy. **(A)** Representative photomicrographs of kidneys sections in each group stained with H&E. **(B)** Representative photomicrographs of kidney sections stained with Sirius red. **(C)** Representative photomicrographs of kidney sections stained with Masson’s trichrome. **(D,E)** Quantitative analysis of interstitial collagen area stained with Sirius red or Masson’s trichrome in the kidneys. **p < 0.01; *n* = 6 in each group; Scale bar: 50 μm.

**FIGURE 8:**
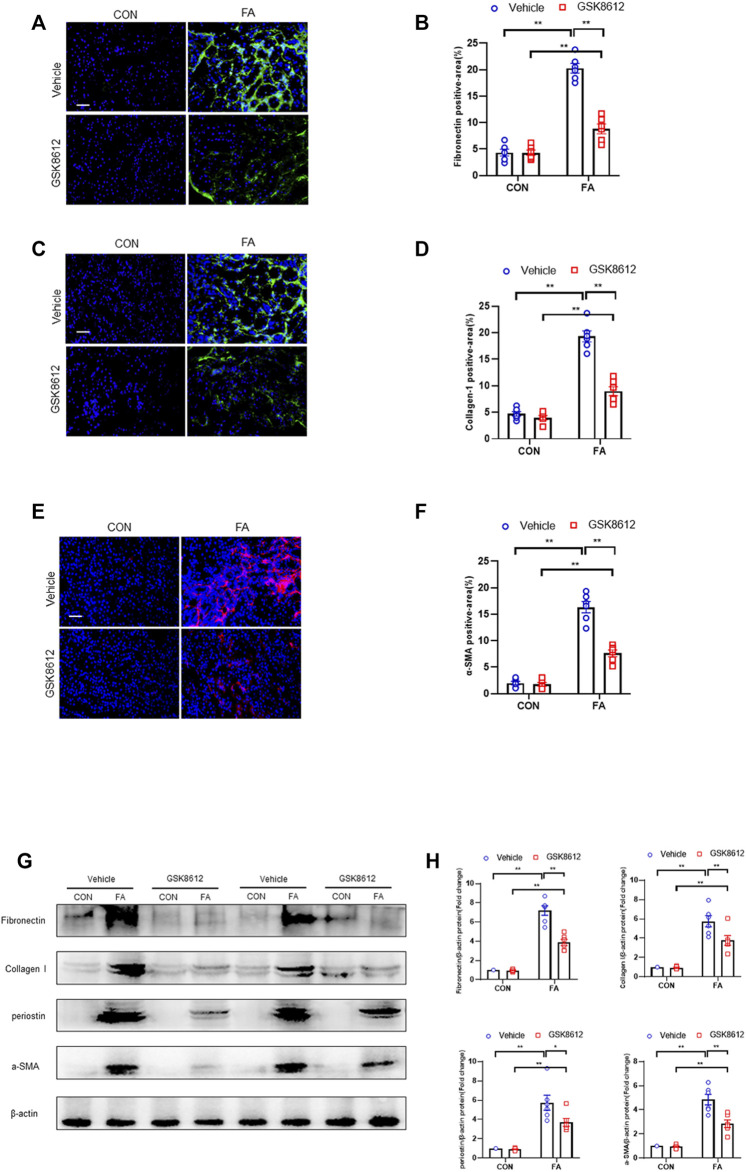
Pharmacological inhibition of TBK1 reduces ECM proteins expression and fibroblasts activation in FA nephropathy. **(A)** Representative immunofluorescence photomicrographs of kidney sections in each group stained for fibronectin (green) and DAPI (blue). **(B)** Quantitative analysis of fibronectin-positive area in the kidneys. **(C)** Representative photomicrographs of kidney sections in each group stained for collagen I (green) and DAPI (blue). **(D)** Quantitative analysis of collagen I-positive area in the kidneys. **(E)** Representative immunofluorescence photomicrographs of kidney sections in each group stained for α-SMA (red) and DAPI (blue). **(F)** Quantitative analysis of α-SMA-positive area in the kidneys. **(G)** Representative western blots show the levels of fibronectin, collagen I, periostin, and α -SMA protein expression in each group. **(H)** Quantitative analysis of fibronectin, collagen I, periostin, and α-SMA protein expression levels in the kidneys. **p < 0.01, *p < 0.05; *n* = 6 in each group; Scale bar: 50 μm.

We next investigated the effect of pharmacological inhibition of TBK1 on the progression of renal fibrosis in FA nephropathy. Our results revealed that GSK8612 treatment significantly reduced the area of collagen deposition in injured kidneys of mice as compared with the controls (Figures 7A–E). In agreement with the results of collagen deposition area, immunofluorescence staining revealed that administration of GSK8612 caused a significant reduction of ECM proteins and α-SMA positive area in kidneys of mice following FA injection (Figures 8A–F). In addition, the injured kidneys of mice with GSK8612 treatment presented lower levels of ECM and α-SMA proteins (Figures 8G,H). These data indicate that pharmacological inhibition of TBK1 attenuates the development of kidney fibrosis in FA nephropathy.

### Pharmacological Inhibition of TBK1 Down-Regulated ECM Proteins and α-SMA Expression and Impairs MMT in Cultured Monocytes

**FIGURE 9:**
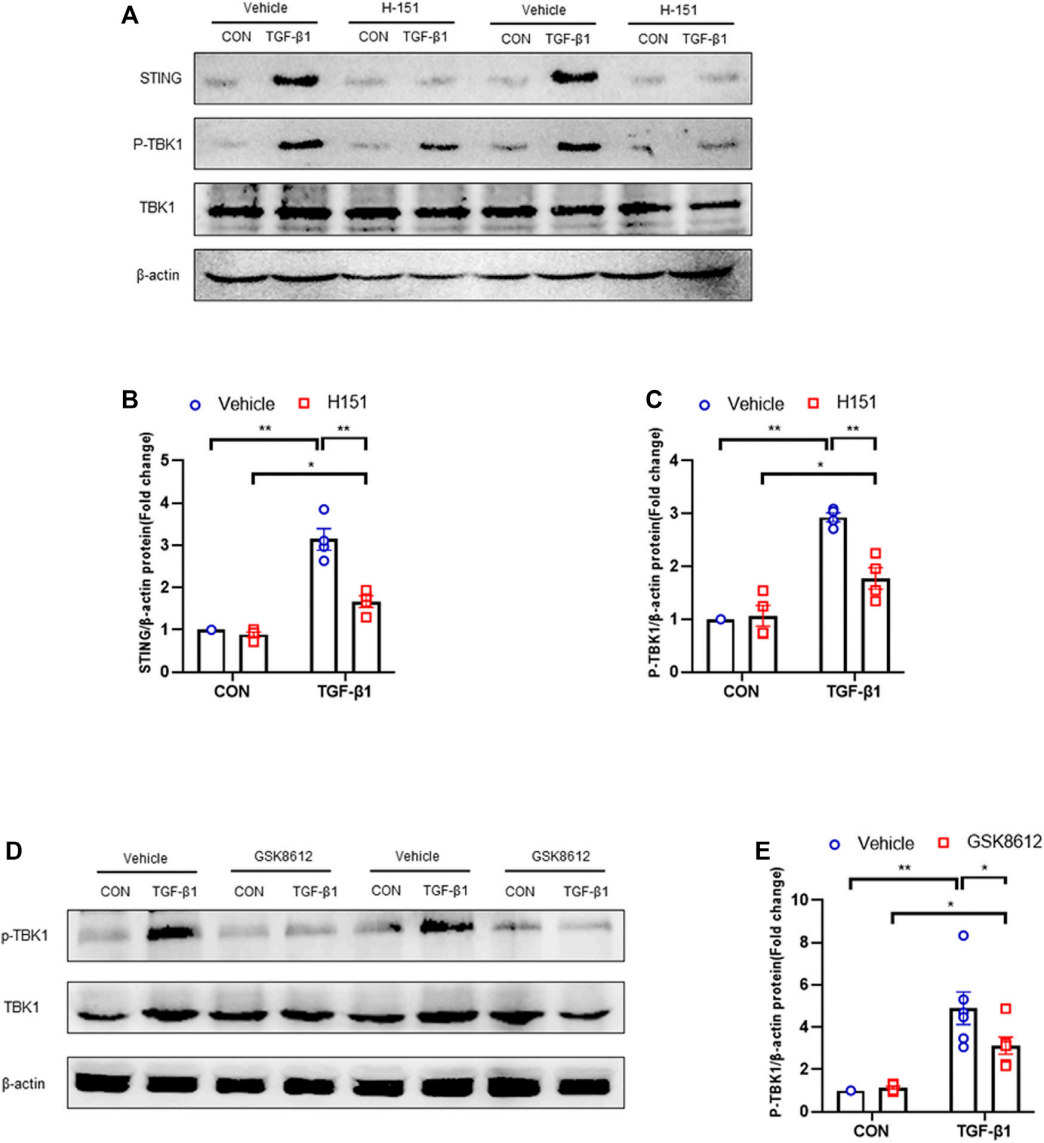
H-151 or GSK8612 treatment inhibits STING/TBK1 signaling activation *in vitro*. **(A)** Representative western blots show STING and TBK1 protein levels in cultured monocytes treated by TGF-β1 or (and) H-151. **(B,C)** Quantitative analysis of STING and TBK1 protein expression. **(D)** Representative western blots show TBK1 protein levels in cultured monocytes treated by TGF-β1 or (and) GSK8612. **(E)** Quantitative analysis of TBK1 protein expression. **p < 0.01, *p < 0.05; *n* = 4–6 in each group.

**FIGURE 10:**
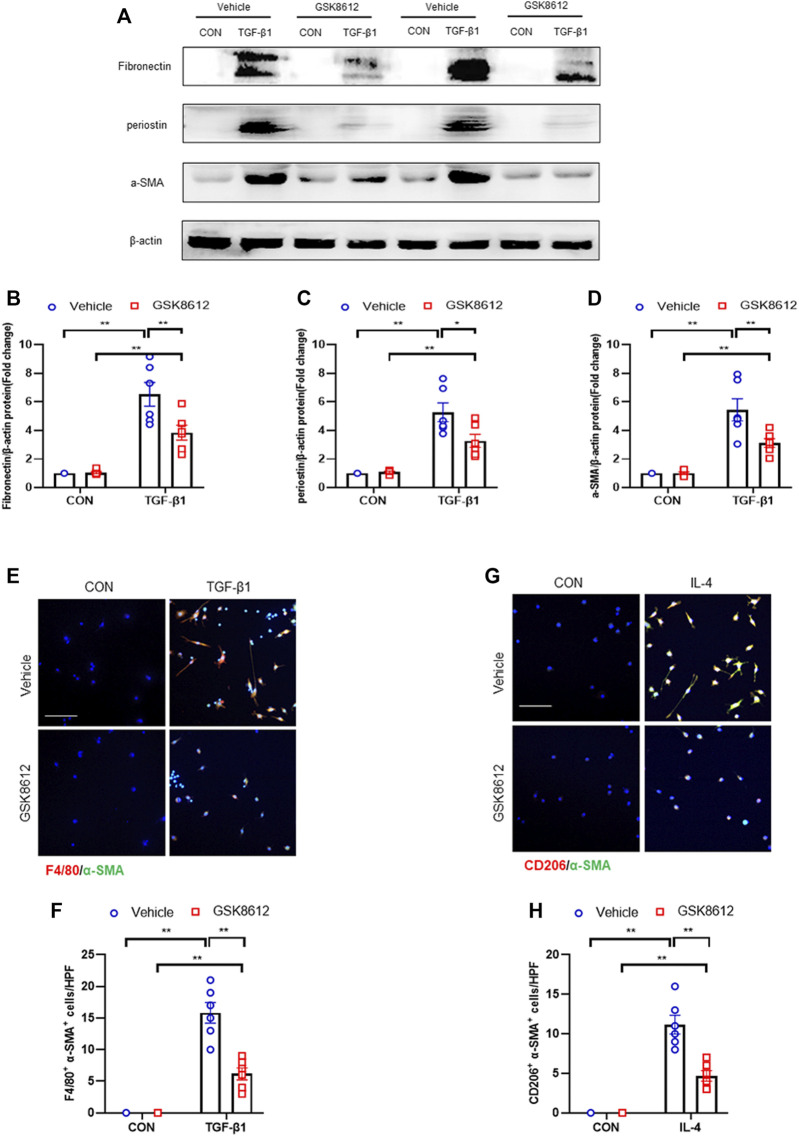
GSK8612 treatment down-regulates ECM proteins and α-SMA expression and impairs MMT *in vitro*. **(A)** Representative western blots show the levels of ECM proteins expression in cultured monocytes treated by TGF-β1 or (and) GSK8612. **(B–D)** Quantitative analysis of ECM proteins expression. **(E)** Representative photomicrographs of cultured monocytes stained for F4/80 (red), α-SMA (green), and DAPI (blue). **(F)** Quantitative analysis of F4/80 and α-SMA dual positive cells. **(G)** Representative photomicrographs of cultured monocytes stained for CD206 (red), α-SMA (green), and DAPI (blue). **(H)** Quantitative analysis of CD206 and α-SMA dual positive cells. **p < 0.01, *p < 0.05; *n* = 6 in each group; Scale bar: 50 μm.

We next investigated if TBK1 inhibition attenuates transition of bone marrow-derived monocytes to myofibroblasts *in vitro*. We showed that TGF-β1 treatment activated STING/TBK1 signaling (Figures 9A,E) and stimulated expressions of ECM and α-SMA proteins of cultured monocytes, which were abolished by GSK8612 treatment (Figures 10A–D). Furthermore, TGF-β1 or IL-4 led to a sharp elevation of the number of F4/80^+^-α-SMA^+^ cells or CD206^+^-α-SMA^+^ cells, respectively. Conversely, GSK8612 treatment markedly reduced the number of F4/80^+^-α-SMA^+^ cells or CD206^+^-α-SMA^+^ cells in cultured monocytes (Figures 10E–H). These data indicates that TBK1 plays an important role in the transition of bone marrow-derived monocytes to myofibroblasts.

**FIGURE 11:**
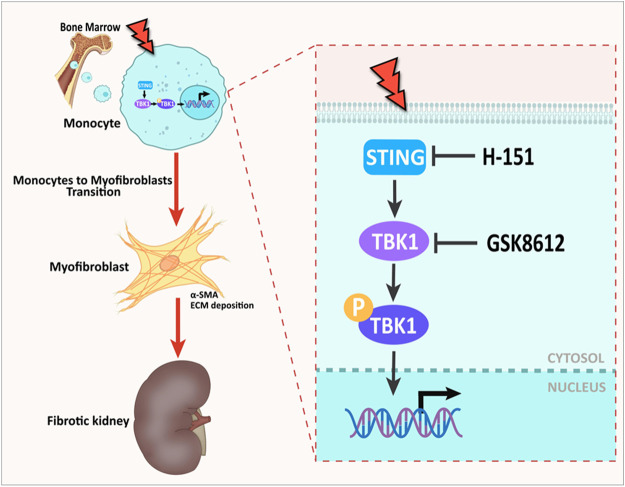
Graphical abstract. The role of STING/TBK1 axis in progressive kidney fibrosis following folic acid stress. Folic acid stress triggers expressions of STING, which subsequently activates TBK1, leading to renal fibrogenesis. Pharmacological inhibition of STING/TBK1 signaling by H-151 or GSK8612 impairs myeloid fibroblasts activation, protects against macrophages to myofibroblasts transition, thus delay the progression of kidney fibrosis.

## Discussion

FA nephropathy is a useful model for studying inflammation and progressive renal fibrosis after acute kidney injury. Our previous studies have shown that FA treatment can cause severe fibrosis and renal dysfunction of mice at the end of 14 days (Liang et al., 2017a; Chen et al., 2021a; Liu et al., 2021b). The activation of myeloid fibroblasts and transition of macrophages to myofibroblasts are proved to be critical factors that contribute to progression of kidneys fibrosis (Jiao et al., 2021a; Jiao et al., 2021b; Liu et al., 2021b). However, the molecular signaling mechanisms that trigger bone marrow-derived fibroblasts activation and promote macrophages to myofibroblasts differentiation are not completely understood. Recently, STING/TBK1 signaling pathway has been highlighted as an important strong immune driver that initiates the release of type I interferon (Yum et al., 2021). Following up-stream signaling stimuli, STING translocates to the perinuclear site and positively regulates activation of TBK1 to enhance the phosphorylation of down-stream targets (Dewi Pamungkas Putri et al., 2019). In the present work, we show that the activity of STING/TBK1 signaling axis is significantly up-regulated in FA nephropathy. STING inhibitor is able to potently suppress the activation of STING/TBK1 signaling in fibrotic kidneys following FA injury. Furthermore, our findings reveal that STING/TBK1 axis exerts a crucial role in renal fibrosis induced by folic acid. We demonstrate that pharmacological inhibition of STING/TBK1 signaling pathway protects against the activation of bone marrow–derived fibroblasts and prevents the transition of macrophages to myofibroblasts in the progression of kidney fibrosis.

STING is deeply related to fibrotic disease, including cardiac fibrosis, pulmonary fibrosis, and liver fibrosis (Luo et al., 2018; Li et al., 2022a; Hu et al., 2022). In general, targeting inhibition of STING has an anti-fibrotic effect (Han et al., 2021). Chung K et al. reported that STING deficiency or STING inhibitor C-176 ameliorated kidney fibrosis (Chung et al., 2019). However, the underlying molecular mechanism for role of STING in renal fibrosis has not been explored. In this study, we performed further investigation to clarify the detailed roles of STING in kidney fibrogenesis. Recently, strong evidences have demonstrated that myeloid myofibroblasts contribute significantly to kidney fibrosis. Bone marrow–derived fibroblast precursors express hematopoietic markers such as CD45 (Liang et al., 2017a). In response to persistent inflammatory stimuli, bone marrow-derived fibroblasts are activated and further differentiate into myofibroblasts (Liang et al., 2017a; Jiao et al., 2021a; Jiao et al., 2021b). Of note, STING has an important role in the regulation of bone marrow-derived macrophages activation (Yan et al., 2018). Thus, we examined whether STING regulates myeloid fibroblasts activation in renal fibrosis in this study. Our findings show that the number of double positive cells of myofibroblasts marker (α-SMA) and myeloid cells marker (CD45) in the damaged kidney of mice was significantly increased, while the number of CD45^+^-α-SMA^+^ double positive cells in injured kidney was decreased profoundly after STING inhibitor treatment. We also reveal that administration of STING inhibitor reduces collagen deposition and delays kidney fibrosis induced by FA insult. These results suggest that STING has an important role in regulating bone marrow-derived fibroblasts activation and the development of renal fibrosis in FA nephropathy.

Emerging data show that macrophages can differentiate directly into myofibroblast-like cells, which is termed macrophages to myofibroblasts transition (Tang et al., 2019). MMT cells express both myofibroblasts marker (α-SMA) and macrophages marker (CD68 or F4/80), which produce a large amount of ECM proteins (Vierhout et al., 2021). MMT is recognized as an important factor that accelerates fibrotic process (Liang et al., 2017a; Feng et al., 2020). STING expression in monocyte-derived macrophages is closed related to the development of liver fibrosis in patients (Wang et al., 2020). Thus, we investigated whether STING plays a role in renal fibrosis by the regulation of MMT. Our findings show that pharmacological inhibition by H-151 impedes the transition of macrophages to myofibroblasts in progressive kidney fibrosis, suggesting STING has a critical role in the modulation of MMT in FA nephropathy. Numerous studies have documented that M2 macrophages promote kidney fibrosis by producing numbers of pro-fibrotic factors and triggers fibroblast activation (Feng et al., 2018; Jiao et al., 2021a; Jiao et al., 2021b; Li et al., 2021). In addition, the cells involved in MMT are mainly M2-like phenotype (Zhang et al., 2016; O'Brien and Spiller, 2021). In this work, we also reveal that administration of FA results in a sharp elevation of the number of CD206^+^-α-SMA^+^ cells in progressive renal fibrosis, while H-151 treatment significantly reduces these cells number. Our results indicate that STING plays an important role in the regulation of M2 macrophages to myofibroblasts transition in development of kidney fibrosis.

Recent data demonstrate that TBK1 has an important role in fibrotic disorder. Inhibition of TBK1 by amlexanox protects against liver fibrosis induced by CCl4 (Zhou et al., 2020). TBK1 knockdown or inhibition significantly ameliorates radiation-induced pulmonary fibrosis and collagen deposition (Qu et al., 2019; Li et al., 2022b). In the current study, we show that STING/TBK1 signal axis is significantly activated. Pharmacological inhibition of STING prevents the activation of TBK1 in injured kidneys or cultured bone marrow-derived monocytes. Therefore, we next observed whether TBK1 inhibition of TBK1 attenuates kidney fibrosis in FA nephropathy. To the best of our knowledge, this is the first time to report that the effect of inhibition of TBK1 on the development of kidney fibrosis. Our findings show that inhibition of TBK1 by GSK8612 prevents ECM accumulation, reduces fibroblasts activation, and delay renal fibrosis progression. Of note, FA injury lead to a sharp increment of the number of CD45^+^-α-SMA^+^ cells in kidneys of mice. In contrast, pharmacological inhibition of TBK1 decreased the number of CD45^+^-α-SMA^+^ cells in injured kidneys. Our findings also reveal that pharmacological inhibition of TBK1 impedes MMT or M2MMT in FA nephropathy. In this study, we show that TBK1 mediates the signaling of STING and plays a critical role in myeloid fibroblasts activation in renal fibrosis. Therefore, we investigated if TBK1 inhibition attenuates the transition of bone marrow-derived monocytes to myofibroblasts *in vitro*. In cultured monocytes, inhibition of TBK1 retards the transition of macrophages to myofibroblasts and down-regulates ECM proteins expression. These data indicates that TBK1 has a crucial role in the activation of myeloid fibroblasts, transition of macrophages to myofibroblasts, and the progression of renal fibrosis in FA nephropathy.

In this study, we adopted a FA nephropathy model of 14 days. Newbury et al. used a chronic FA nephropathy model (12 weeks) and revealed that inhibition of Kirsten-Ras reduces fibrosis and protects against renal dysfunction (Newbury et al., 2019). In this respect, further investigation is required for assessing whether pharmacological inhibition of STING/TBK1 has a role in a longer term of FA nephropathy model. The pathogenesis of FA nephropathy is complicated. It is reported that RIPK3 inhibition or RIPK3 deficiency attenuates renal fibrosis via the regulation of the NLRP3 inflammasome in a mouse model of FA nephropathy (Shi et al., 2020). Whether STING/TBK1 axis plays a role in FA nephropathy through a similar signaling mechanism also needs further investigation.

There are limitations in our study. Firstly, our study is limited to animal experiment. FA model simulates drug-induced crystal nephropathy. The kidney pathology following FA injury is similar with human AKI from drugs or toxins (Ortiz et al., 2015). On the other hand, FA model is useful for studying AKI-to-CKD transition but no clinical correlation by now (Aparicio-Trejo et al., 2020). Moreover, suitable biomarkers for STING/TBK1 pathway are still lack. In addition, whether gender or age affects the experimental results requires further investigation.

Taken together, STING/TBK1 signaling contributes to renal fibrosis by the regulation of myeloid fibroblasts activation and marcophages to myofibroblasts transition. Our study highlights a novel mechanism of STING/TBK1 in the pathogenesis of renal fibrogenesis and provides a therapeutic potential for STING/TBK1 inhibitors to treat fibrotic kidneys diseases.

## Data Availability

The original contributions presented in the study are included in the article/supplementary material further inquiries can be directed to the corresponding authors.

## Abbreviations

AKI, acute kidney injury; CKD, chronic kidney disease; FA, folic acid; ECM, extra-cellular membrane; CON, control; FN, fibronectin; COL I, collagen I; α-SMA, α-smooth muscle actin; GAPDH, glyceraldehyde-3phosphate dehydrogenase; MMT, macrophages-to-myofibroblasts transition.
